# South London Hospital

**Published:** 1913-11-15

**Authors:** 


### SOUTH LONDON HOSPITAL.

The long list of vice-presidents of the South London Hospital for Women now includes the Duchess of Marlborough, the Duchess of Sutherland, the Duchess of Westminster, Muriel Viscountess Helmsley, Lord and Lady Tennyson, and the Hon. Mrs. Eric Chaplin. The hospital, shortly to be erected at Clapham Common, is to be entirely officered by women doctors; a woman secretary is now, we believe, in request. The out-patient department opened in April last at 88-90 Newington Causeway, S.E., is finding it difficult to cope with the increasing numbers of women who come for treatment, even after the almoner has weeded out unsuitable cases.

